# Comparison of clinical outcomes in peripartum cardiomyopathy and age-matched dilated cardiomyopathy: A 15-year nationwide population-based study in Asia

**DOI:** 10.1097/MD.0000000000006898

**Published:** 2017-05-12

**Authors:** Cheng-Hui Lu, Wen-Chen Lee, Michael Wu, Shao-Wei Chen, Jih-Kai Yeh, Chun-Wen Cheng, Katie Pei-Hsuan Wu, Ming-Shien Wen, Tien-Hsing Chen, Victor Chien-Chia Wu

**Affiliations:** a Division of Cardiology, Chang Gung Memorial Hospital, Linkou Medical Center, Taoyuan City, Taiwan; b Division of Cardiology, Weill Cornell Medical Center, New York; c Department of Cardiothoracic and Vascular Surgery, Chang Gung Memorial Hospital, Linkou Medical Center; d Department of Infectious Diseases, Chang Gung Memorial Hospital, Linkou Medical Center; e Department of Rehabilitation, Chang Gung Memorial Hospital, Linkou Medical Center; f College of Medicine, Chang Gung University, Taoyuan City, Taiwan; g Department of Cardiology, Chang Gung Memorial Hospital, Keelung, Taiwan.

**Keywords:** dilated cardiomyopathy, outcome, peripartum cardiomyopathy

## Abstract

Peripartum cardiomyopathy (PPCM), often classified as a form of dilated cardiomyopathy (DCM), is the myocardial dysfunction that occurs in late pregnancy and through the first few postpartum months.

The aim of this study is to investigate the differences in the clinical outcomes of PPCM and DCM.

Electronic medical records from 1997 to 2011 were retrieved from the Taiwan National Health Insurance Research Database. Patients with PPCM were compared with age- and clinical characteristics-matched patients with DCM. Primary outcomes were 1- and 3-year heart failure (HF) readmission, cardiac death, all-cause mortality, and major adverse cardiovascular events. Secondary outcomes were myocardial infarction, new onset of dialysis, heart transplant, and cerebrovascular accident. Follow-up period was divided into “within the first year” and “after the first year.”

A total of 527,979 patients (253,166 females) were hospitalized with a principal diagnosis of HF during 1997 to 2011 period. After excluding patients aged <18 and >50 years, patients with other forms of HF, and those with a history of cerebrovascular accidents or coronary artery disease, 797 patients with PPCM and 1267 patients with DCM were evaluated. Propensity score matching yielded 391 patients in each group. Patients with DCM had a significantly worse prognosis compared to those with PPCM for all primary and secondary outcomes at the 1- and 3-year follow-ups. After 1 year, the HF readmission rate did not significantly differ between the 2 diseases, suggesting that HF medications should be aggressively instituted in patients with PPCM.

This is the first study to directly compare the clinical outcomes between age-matched patients with PPCM and DCM. Patients with PPCM had a significantly better prognosis across all cardiovascular endpoints compared to patients with DCM.

## 1 Introduction

Peripartum cardiomyopathy (PPCM) is the development of heart failure (HF) in pregnant women at the time of or in the months following childbirth, and this condition can be ominous. The National Heart, Lung, Blood Institute and Office of Rare Diseases have defined PPCM as follows: the development of HF in the last month of pregnancy or within 5 months of delivery, the absence of a determinable etiology for HF, the absence of demonstrable heart disease before the last month of pregnancy, and echocardiographic evidence of left ventricular (LV) systolic dysfunction.^[1–3]^ Recently, the Working Group on PPCM of the Heart Failure Association of the European Society of Cardiology (ESC) has proposed encompassing HF secondary to idiopathic LV dysfunction occurring toward the end of pregnancy or in the months following delivery, where no other cause of HF is found, with the majority of HF diagnosed during the third trimester of pregnancy.^[4–6]^ The pathophysiology of PPCM is closely timed with the process of pregnancy and delivery; however, the etiology of PPCM is uncertain and is attributed widely to myocarditis, autoimmunity, excessive hemodynamic load, hormonal imbalance, nutritional deficiency, and genetic mutation.^[7]^ Although some experts disagree, current medical literature categorizes PPCM as a type of dilated cardiomyopathy (DCM) initiated during pregnancy, and this condition presents as idiopathic chamber dilatation and LV dysfunction.^[8,9]^

Patients with PPCM typically present in their early 30s, but patients with DCM usually present later in life, unless a familial or genetic predisposition exists. Patients with PPCM have a relatively high LV systolic function recovery rate of 20% to 60% and mortality rate of 11% to 32% at final follow-up.^[10–13]^ By contrast, patients with DCM have a decreasing survival rate with 90% at 1 year, 50% at 5 years, and 33% at 10 years, although improved survival has been noted over the recent decade in a Japanese study.^[14–16]^ Recent advances in genetics have indicated that PPCM shares genetic susceptibility of the titin gene with both familial and sporadic DCM.^[17]^ The reports of PPCM and DCM cases within families further suggest an overlap in the etiology of the 2 diseases.^[18]^ However, less is known on the differences between the 2 conditions.

In this national population-based study, we used the data of a 15-year cohort of female patients admitted with HF, which were provided by the Taiwan National Health Insurance (NHI) Research Database (NHIRD), to compare the natural courses and clinical outcomes between patients with PPCM and DCM.

## 2 Methods

### 2.1 Study patients

The Taiwan NHI Program was started in 1995 and covers 99.5% of the 23 million residents of Taiwan.^[19]^ The NHIRD contains the data of all inpatient and outpatient services, diagnoses, prescriptions, examinations, operations, and expenditures, and these data are updated biannually. More than 95% of Taiwan's 23 million residents are Han Chinese; therefore, our study sample can be considered to be ethnically uniform. The Institutional Review Board of Chang Gung Memorial Hospital Linkou Branch approved this study.

Through a search of the medical records stored in the NHIRD between 1 January 1997 and 31 December 2011, we retrieved the data of all patients admitted for HF, and the data of female patients were marked for a further study. On the basis of the latest definition of PPCM by the ESC Working Group,^[4–6]^ PPCM was identified in women hospitalized with HF from the last trimester of pregnancy until 5 months after delivery. HF was initially screened for using the International Classification of Diseases, 9th Revision, Clinical Modification (ICD-9-CM) codes for HF (428.xx), primary and secondary cardiomyopathies (425.4, 425.9), PPCM (674.5), and myocarditis (429.0). Women who met these criteria underwent a detailed review of their medical history to confirm the diagnosis of PPCM. PPCM was confirmed if the following criteria were satisfied: no previous diagnosis of HF, diagnosis of HF occurring in the last trimester pregnancy until 5 months after delivery, and no other cause of HF could be identified.

The main limitation of studies using data derived from the NHIRD is the unavailability of the detailed reports of examinations, such as ejection fractions, in the retrievable database. Nevertheless, the diagnosis of HF recorded in the NHIRD has been previously validated against the gold standard, namely hospital electronic medical records (EMRs). The diagnosis of various diseases recorded in the NHIRD, including hypertension, diabetes mellitus, HF, and acute renal failure, has been validated against hospital EMRs, with high accuracy. For example, the diagnosis of hypertension in the NHIRD had 97% sensitivity and 95% positive predictive value (PPV) against hypertension in EMRs. Similarly, diabetes had 98% sensitivity and 95% PPV, HF had 99% sensitivity and 99% PPV, and acute renal failure had 92% sensitivity and 100% PPV.^[20]^

As there was no single code available for DCM in ICD-9-CM, we first excluded patients with a history of prior pregnancy and cardiomyopathy diagnoses (425.0–425.3 and 425.5–425.9). Within the ICD-9-CM code 425.4 (with other primary cardiomyopathies), DCM was identified by excluding storage disease-related cardiomyopathies. In addition, among both PPCM and DCM patients, those with a history of coronary artery disease (including a history of myocardial infarction, percutaneous intervention, and coronary artery bypass graft), cerebrovascular accidents, and HF were excluded. Age- and comorbidity-matched DCM patients were then selected, and the long-term outcomes were compared with those in PPCM patients.

### 2.2 Covariate and study outcomes

To effectively compare 2 groups of patients whose ages of presentation typically differed, we matched the clinical characteristics of patients with DCM to those of patients with PPCM because excess number of patients with DCM were present. In addition to age, patients with coexisting conditions, such as hypertension, diabetes mellitus, and hyperlipidemia, which are precursors to ischemic heart disease but are not yet established coronary artery disease, were matched by propensity scores.

The medical records in the NHIRD lists the primary diagnoses of the patients recorded during admission. The definitions of cardiovascular death in the NHIRD meet the criteria of the Standardized Definitions for End Point Events in Cardiovascular Trials draft published by the US Food and Drug Administration. Death was defined as the withdrawal of the patient from the NHI Program. The causes of death were defined according to the primary discharge diagnosis of hospitalization within 3 months before death. The primary outcomes were all-cause mortality, cardiac death, HF readmission, and major adverse cardiovascular events (MACE). MACE included myocardial infarction, cerebrovascular accidents, HF readmission, heart transplant, and cardiac death. The secondary outcomes were myocardial infarction, new onset of dialysis, heart transplant, and cerebrovascular accidents.

### 2.3 Statistical analysis

We compared the patients’ clinical characteristics, including examinations, interventions, and medications, between the study groups of PPCM and DCM by using the *χ*^2^ test for categorical variables and independent sample *t* test for continuous variables. The cumulative incidence of time-to-event outcome (i.e., all-cause mortality) during the prespecified periods (i.e., 1 and 3 years) was compared between the study groups by using a Cox proportional hazards model adjusted for propensity scores. All statistical analyses were performed using commercial software (SAS V.9.4, SAS Institute, Cary, NC).

## 3 Results

### 3.1 Study population

**Figure 1:**
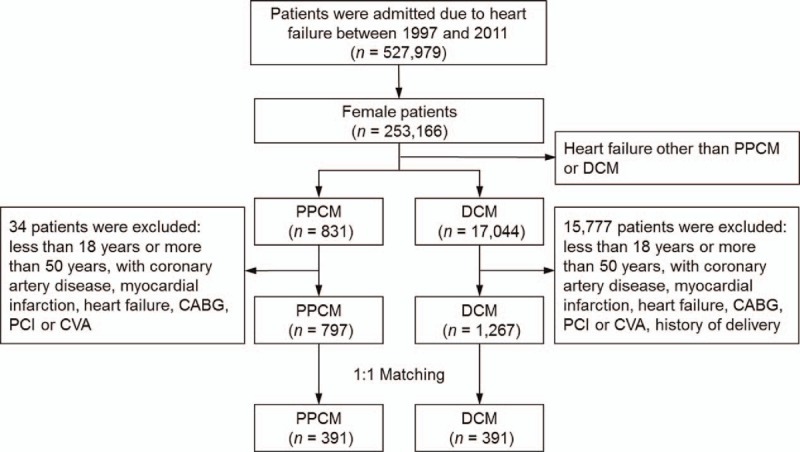
Study design and screening criteria flowchart for the inclusion of PPCM and DCM patients. CABG = coronary artery bypass surgery, CVA = cerebrovascular accident, DCM = dilated cardiomyopathy, PCI = percutaneous coronary intervention, PPCM = peripartum cardiomyopathy.

The data of 527,979 patients admitted because of HF between 1997 and 2011 were retrieved from the NHIRD; among these patients, 253,166 were females. After excluding patients with HF other than PPCM or DCM, aged <18 and >50 years, and with a history of coronary artery disease or cerebrovascular accidents, 797 patients with PPCM and 1267 patients with DCM were evaluated. Propensity score matching by age, history of hypertension, diabetes mellitus, and hyperlipidemia yielded 391 patients each in the PPCM and DCM groups (Fig. 1). A comparison of the mean ages revealed that patients with PPCM were 10 years younger than those with DCM (Table 1). After 1:1 matching, the mean ages of patients with PPCM and DCM were 32.9 ± 5.8 and 32.5 ± 7.6 years, respectively, and no significant differences in age, history of hypertension, diabetes mellitus, and hyperlipidemia were observed between the 2 groups.

**Table 1:**
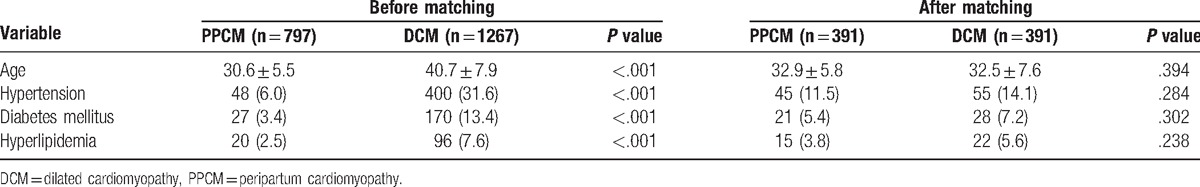
Clinical characteristics of the study patients.

### 3.2 Clinical characteristics

Table 2 shows the findings of patients with PPCM and DCM after matching for interventions, medications, inotropic agents, and in-hospital outcomes during the index admission. Patients with PPCM received less prescriptions of aspirin and angiotensin-converting enzyme inhibitors (ACEi)/angiotensin receptor blockers (ARB) because ACEi/ARB are pregnancy category D drugs. By contrast, a higher percentage of critical care medications, specifically, inotropics, were used in patients with PPCM. No significant differences were observed between the study groups in their in-hospital outcomes of ICU stay, hospitalization duration, and in-hospital death.

**Table 2:**
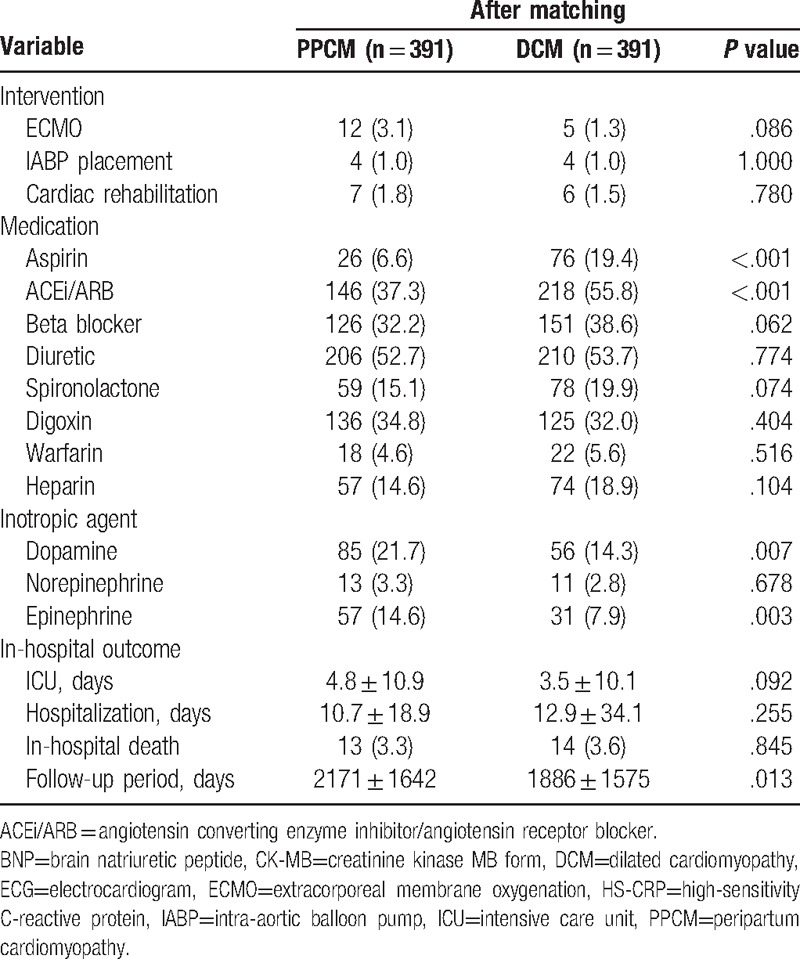
Intervention, medication, and outcome during the admission.

### 3.3 1- and 3-year outcomes

Table 3 lists the results of primary outcomes, namely all-cause mortality, cardiovascular death, HF readmission, and MACE, and secondary outcomes, namely myocardial infarction, new-onset dialysis, heart transplant, and cerebrovascular accidents. At the end of 1 and 3 years of follow-up, the patients with PPCM had significantly more favorable results for all primary and secondary outcomes.

**Table 3:**
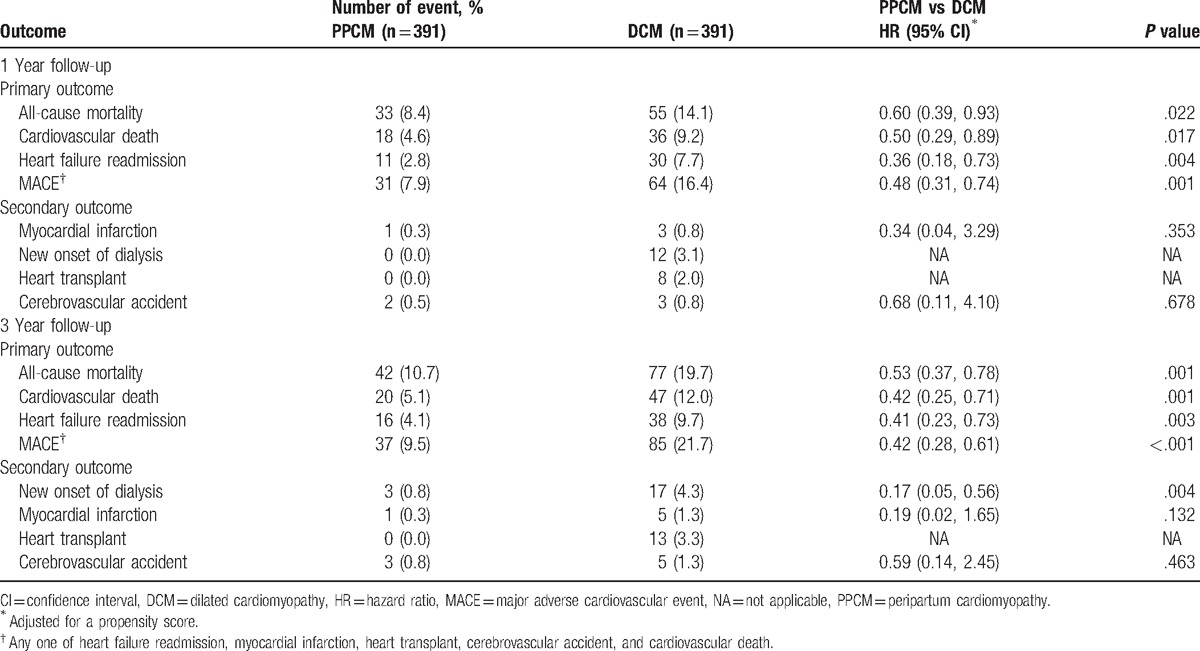
Long-term outcome after 1 year during the PPCM and DCM admission.

**Figure 2:**
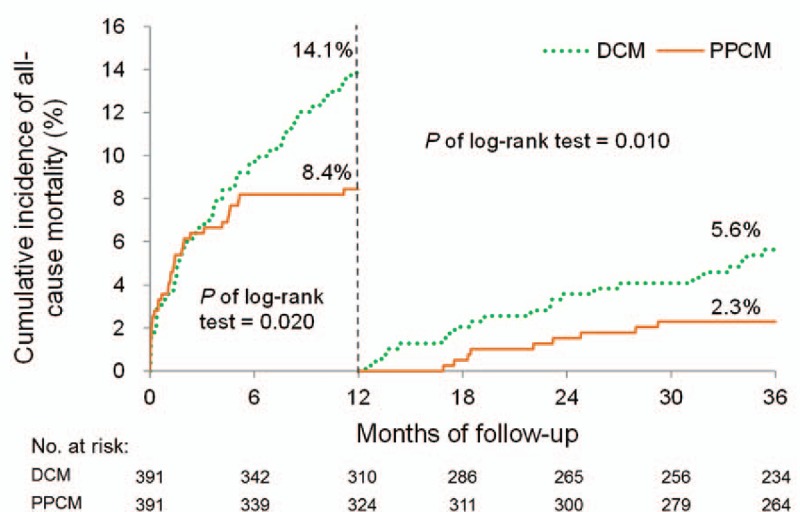
Cumulative incidence of all-cause mortality. Patients with PPCM had significantly better outcomes than did those with DCM at the 1-year follow-up. This difference remained significant at the 3-year follow-up. DCM = dilated cardiomyopathy; PPCM = peripartum cardiomyopathy.

**Figure 3:**
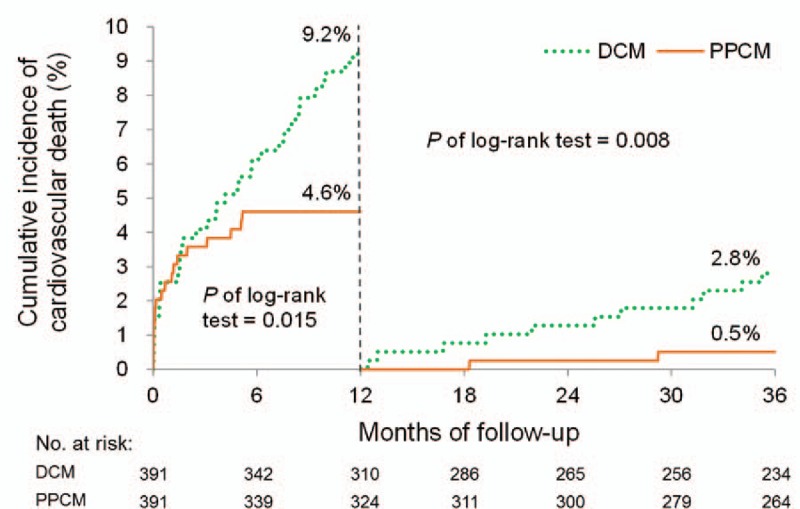
Cumulative incidence of cardiovascular death. Patients with PPCM had significantly better outcomes than did those with DCM within 1-year follow-up. This difference remained significant after the 1-year follow-up. DCM = dilated cardiomyopathy; PPCM = peripartum cardiomyopathy.

**Figure 4:**
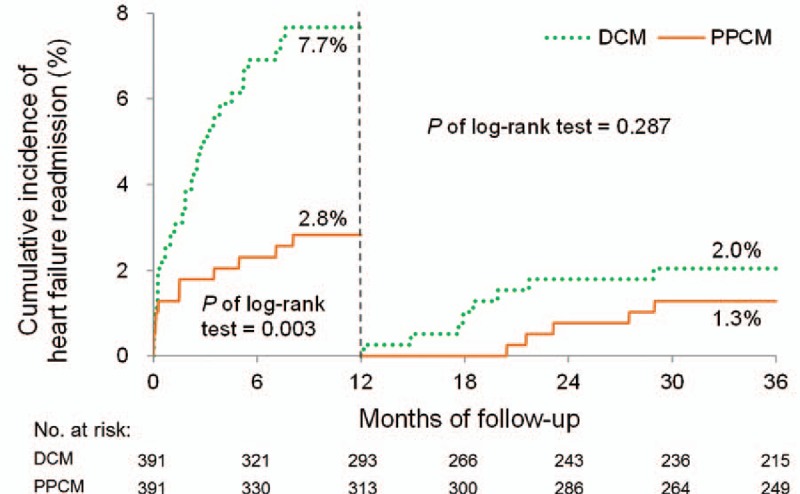
Cumulative incidence of all-cause mortality. Patients with PPCM had significantly better outcomes than did those with DCM within 1-year follow-up. This difference ceased to be significant after the 1-year follow-up. DCM = dilated cardiomyopathy; PPCM = peripartum cardiomyopathy.

**Figure 5:**
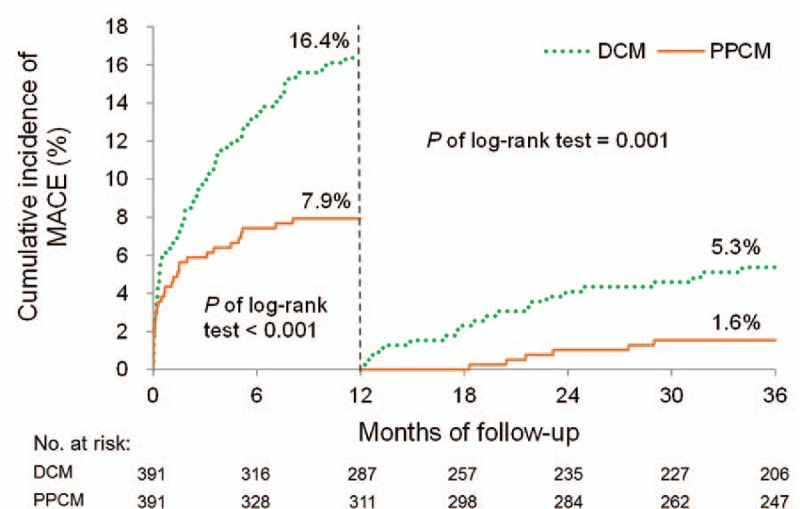
Cumulative incidence of MACE. Patients with PPCM had significantly better outcomes than did those with DCM within 1-year follow-up. This difference remained significant after the 1-year follow-up. DCM = dilated cardiomyopathy; MACE = major adverse cardiovascular events; PPCM = peripartum cardiomyopathy.

The cumulative incidence plots revealed that the patients with PPCM had significantly better 1-year outcomes in terms of all-cause mortality, cardiac death, HF readmission, and MACE, as previously described (Figs. 2–5). Patients with PPCM still demonstrated significantly better prognosis for all-cause mortality, cardiac death, and MACE (*P* = .010, .008, and .001, respectively) during the second and third years of follow-up. No difference was observed in the risk of HF readmission between the groups during the second and third years of follow-up (*P* = .287).

## 4 Discussion

Our study has several findings: this is the first study to directly compare the clinical outcomes between PPCM and DCM; cardiovascular morbidity and mortality of patients with PPCM were consistently and significantly less than those of patients with DCM; and although earlier studies have considered PPCM a form of DCM, PPCM has a specific time frame and circumstances in which the myocardial dysfunction develops, which ultimately results in a clinical picture distinct from that of DCM.

### 4.1 Previous studies

The national incidence of PPCM ranges from 1 in 1000 to 1 in 4000 in the United States, with Whites, African–Americans, Hispanics, and Asian–Americans having incidences of 1 in 4075, 1 in 1421, 1 in 9861, and 1 in 2675 deliveries, respectively.^[9,21]^ A higher incidence of PPCM in 1 in 300 live births was reported in Haiti.^[22]^ Estimates of the incidence and prevalence of idiopathic DCM were originally based on a study from 1975 to 1984 in Olmstead County, Minnesota, United States, in which 46 individuals were identified with idiopathic DCM, indicating an age- and sex-adjusted incidence of 6.0 per 100,000 person-years and a prevalence of 36.5 in 100,000 of the population (i.e., ∼1 in 2700 individuals).^[23]^ However, DCM accounts for 25% of all cases of HF and is responsible for nearly 50,000 hospitalizations and 10,000 deaths each year in the United States.^[24]^

PPCM is often considered as DCM unmasked during pregnancy, with subtle LV dysfunction exacerbated by concurrently increased hemodynamic stress and fluid overload in these women. However, in certain patients with PPCM, overt LV failure with an LV ejection fraction of 10% to 30% on presentation has been observed. A previous study of PPCM reported a 50% recovery rate of LV dysfunction during follow-up (mostly within 6 months), and 1 prospective study of patients with PPCM in the United States described a recovery rate of up to 72%.^[2,25]^ By contrast, in a study on the frequency of recovery and relapse in 188 patients with DCM who were followed up for 50 ± 31 months, 41% patients improved, with 64% exhibiting sustained improvement and the remaining 36% relapsed in further follow-ups of 36 ± 25 months.^[26]^

Several hypotheses could also explain the observed clinical similarity between the 2 investigated diseases, including similarities in the altered myocardial structure and vasculature. Studies on the genetics of sarcomere protein have suggested that the titin isoform plays a role in the ability of the heart to adapt and respond to stretch, through a mechanism known as the Frank–Starling law. Up to 25% familial DCM and 18% sporadic DCM patients exhibited a titin mutation.^[27]^ In a study that enrolled patients with PPCM to investigate the truncating variants of the titin gene, ∼10% patients shared such genetic mutations.^[17]^ However, little is known regarding the cause of the pathogenic mutation, and titin gene variations are not always disease triggering but rather disease modifying.^[27]^

In the vascular system, disturbances in the ubiquitin–proteasome system lead to elevated asymmetric dimethylarginine (ADMA) levels, which cause reduction in nitric oxide availability.^[28]^ Increased levels of ADMA have been noted in patients with PPCM and DCM, and a decreased l-arginine/ADMA ratio is a predictor of mortality in DCM.^[29]^ In addition, in late pregnancy, antiangiogenic factors are secreted to inhibit vascular endothelial growth factor signaling. The significantly higher increase in antiangiogenic factors observed in patients with (pre)eclampsia has been associated with the development of PPCM, with reduced capillary density observed in the postpartum phase in these patients.^[30,31]^

### 4.2 Current study

Our results support the notion that differential pathophysiologies are responsible for the development of these 2 apparently similar cardiomyopathies secondary to the chamber dilatation of ventricular dysfunction. The clinical consequences of cardiovascular compromise, including hemodynamic instability, myocardial ischemia, abnormal coagulation cascade, circulatory insufficiency, severe contractile failure, and mortality, were evident in the sequelae of HF readmission, myocardial infarction, cerebrovascular accidents, new-onset dialysis, heart transplant, and cardiovascular death observed in these patients. From a clinical perspective, our results revealed that patients with PPCM had significantly less cardiovascular events during the short-term 1-year follow-up for all primary and secondary endpoints. The patients with PPCM continued to demonstrate a significantly better prognosis during the 3 years of follow-up (Table 3).

The cumulative incidence was categorized into “within 1 year” and “after 1 year” of follow-up, and the results revealed that patients with PPCM had significantly better primary outcomes, except for HF readmission, during the second and third years of follow-up (Figs. 2–5). Although the medical management of PPCM is similar to that of other forms of systolic HF, physicians are less inclined to use certain drugs, such as ACEi/ARB, which have been associated with increased teratogenicity and fetal loss in potential or de facto childbearing women. Following the same logic, many standard evidence-based HF medications belong to pregnancy category D and are thus less prescribed in patients with PPCM (Table 2). Consequently, patients with PPCM are presumably less protected and treated for HF and HF decompensation.

The physiological adaptations occurring during pregnancy are often attributed to the increased blood volume and red blood cell mass, leading to 15% to 30% increased heart rate, 15% to 25% increased stroke volume, and 20% to 50% increased cardiac output. These hemodynamic changes in the cardiovascular system start during second trimester and peak during the third trimester.^[32,33]^ The myocardium then undergoes eccentric hypertrophy with chamber enlargement, which is reversed postpartum.^[34]^ Although patients with DCM also have ventricular dilatation, such volume-overloading effects are not the primary insult that causes potentially deteriorating myocardial function.

In patients with PPCM, oxidative stress plays a central role in disease pathogenesis. The vasculohormonal hypothesis states that STAT3 has a role in cardiomyocyte protection from reactive oxygen species (ROS). The loss of STAT3 and increased ROS trigger the secretion of cathepsin D, which cleaves prolactin into a 16-kDa fragment, resulting in cell death in PPCM.^[35]^ Prolactin secretion is normally tightly controlled by a negative-feedback mechanism and is inhibited by dopamine. The activation of placental lactogen during pregnancy keeps prolactin secretion low during early and mid-pregnancy. Despite the continued presence of placental lactogen, dopamine secretion is reduced during late pregnancy, in addition to the insensitivity of the feedback loop, allowing a large nocturnal surge of prolactin during the night before parturition.^[36]^ Elevated prolactin levels are maintained through breastfeeding but subside in the months following weaning.

In summary, our results revealed that patients with PPCM had significantly better prognosis than did patients with DCM across all cardiovascular endpoints at the 1- and 3-year follow-ups. After 1 year, patients with PPCM did not have significantly different HF readmission rate compared with patients with DCM, indicating the possible role of aggressive standard HF treatment in these patients.

## 5 Limitations

Epidemiologic data from the NHIRD has several limitations. First, data of the main criteria for the diagnosis of PPCM using LV ejection fraction were not available. Nevertheless, as mentioned in Section 2, the diagnosis of HF described in the NHIRD has 99% sensitivity and 99% PPV against the gold standard EMRs. Second, the use of ICD-9-CM codes for patient screening may have caused some cases of incorrectly coded conditions to be missed. Third, in the DCM pool, the patients with more severe disease may have been selected, because DCM usually presents at an older age. Finally, because our study sample had a homogenous ethnic background, the application of our results to other populations requires interpretation in the appropriate context.

## 6 Conclusions

Our study of PPCM is the first and largest study to directly compare the clinical outcomes between PPCM and DCM. Patients with PPCM exhibited significantly better outcomes than did those with DCM at the 1- and 3-year follow-ups. After 1 year, the HF readmission rate did not significantly differ between the 2 study groups, suggesting that HF medications should be aggressively instituted in patients with PPCM.
